# The effects of cluster-set resistance training on mental health and sleep quality in sedentary young women: protocol for a randomized controlled trial

**DOI:** 10.3389/fpsyg.2026.1846281

**Published:** 2026-06-05

**Authors:** Ming Xiang, Haonan Wang, Yinghong Dai, Liang Yu

**Affiliations:** 1 Department of Exercise Physiology, School of Sport Science, Beijing Sport University, Beijing, China; 2 Engineering Research Center of Strength and Conditioning Training Key Core Technology Integrated System and Equipment of Ministry of Education, Beijing Sport University, Beijing, China; 3 Department of Neurosurgery, Xiangya Hospital, Central South University, Changsha, China

**Keywords:** cluster set, mental health, randomized controlled trial, resistance training, sedentary young women

## Abstract

**Background:**

Mental health and sleep problems are highly prevalent among sedentary young women and substantially impair their well-being. Resistance training has shown promise in improving mental health and sleep outcomes. However, traditional set structures often induce high levels of fatigue and perceived exertion, which may reduce exercise tolerability and compromise sustained participation. By redistributing rest within sets, cluster-set resistance training may attenuate fatigue while preserving comparable training stimuli, thereby potentially enhancing benefits for mental health and sleep.

**Methods:**

This single-blind, parallel-group randomized controlled trial will recruit sedentary female college students. Participants will be randomly allocated (1:1:1) to (1) a non-exercising control group (CON), (2) traditional resistance training (TR), or (3) cluster-set resistance training (CS). The intervention will last 8 weeks (3 supervised sessions/week for TR and CS). Training exercises, weekly load progression, and total rest time per exercise will be identical between TR and CS; only set structure and the distribution of rest intervals will differ. Outcomes will be assessed at baseline, week 4, and week 8. The co-primary outcomes are DASS-21 total score and PSQI global score. Secondary outcomes include POMS total mood disturbance, actigraphy-derived sleep parameters, maximal dynamic strength, anthropometrics and body composition, and blood biomarkers (high-sensitivity C-reactive protein, cortisol, and serotonin).

**Discussion:**

This trial will evaluate whether TR and CS improve mental health and sleep compared with CON, and whether rest-redistribution cluster sets provide additional benefits over traditional set structures under comparable overall training stimuli. The findings may inform the design of future supervised resistance-training interventions and longer-term trials for sedentary young women.

**Clinical trial registration:**

This trial was registered at the China Clinical Trials Registration Center (www.chictr.org.cn), registration number is ChiCTR2500110077.

## 1 Introduction

Mental health problems are a major public-health concern in university populations. The university years coincide with a peak period for the onset of common mental disorders, and cross-national data indicate that roughly one-third of first-year students screen positive for at least one diagnostic and statistical manual of mental disorders mood, anxiety, or substance use disorder (Auerbach et al., 2018). Depressive symptoms are also highly prevalent among university students, including Chinese university students (Ibrahim et al., 2013; Lei et al., 2016). Sleep problems represent another highly prevalent and clinically important concern; a recent meta-analysis estimated that nearly half of undergraduate students experience insomnia symptoms (Spyridonidis et al., 2025). Importantly, mental health and sleep should not be viewed as isolated outcomes. Psychological distress can impair sleep, whereas insomnia and related sleep disturbances increase the risk of subsequent mental disorders, supporting a bidirectional relationship between the two domains (Lo Martire et al., 2020; Hertenstein et al., 2019; Baglioni et al., 2011). Together, poor mental health and disturbed sleep can compromise emotional functioning, daily performance, academic engagement, and overall well-being (Nissen et al., 2021). These concerns are particularly relevant to sedentary young women for several reasons. First, women generally show a higher burden of depressive symptoms and insomnia than men, making psychological distress and sleep disturbance especially salient outcomes in female samples (Salk et al., 2017; Zhang and Wing, 2006). Moreover, in young women, menstrual-related symptoms and hormonal fluctuations may contribute to variability in mood and sleep, which highlights the need for careful assessment standardization (Baker and Driver, 2007). Additionally, prolonged sedentary behavior is common among female college students and is associated with poorer mental health and sleep outcomes (Zhang et al., 2022). Therefore, sedentary young women represent a focused and clinically meaningful subgroup for testing feasible exercise-based interventions.

Exercise is increasingly recognized as a promising non-pharmacological strategy for improving both mental health and sleep. Accumulating evidence suggests that regular physical activity can reduce symptoms of anxiety and depression and can also improve subjective and objective sleep outcomes (Gordon et al., 2020; O'Sullivan et al., 2023; Cunha et al., 2024; Herring and Meyer, 2024; Reid et al., 2010). These findings provide a strong rationale for lifestyle-based interventions aimed at enhancing well-being in young adults. Among the available exercise modalities, resistance training deserves particular attention. Beyond its musculoskeletal benefits, resistance training has shown potential to alleviate depressive and anxiety symptoms and improve sleep-related outcomes across populations (Gordon et al., 2020; O'Sullivan et al., 2023; Cunha et al., 2024; Herring and Meyer, 2024; Reid et al., 2010). Resistance training is also highly adaptable to supervised settings because exercise selection, external load, training volume, and progression can be prescribed and standardized with relative precision, making it both feasible and scalable in campus-based and novice populations, including young women.

However, the benefits of resistance training depend not only on participation but also on how the training stimulus is organized. In addition to frequency, intensity, and total volume, set structure is an important yet underexplored programming variable (Tufano et al., 2017). Traditional set configurations typically require repetitions or hold to be completed continuously until the prescribed work is achieved, which can induce substantial neuromuscular and perceptual fatigue (Izquierdo et al., 2006; Hooper et al., 2014; Gonzalez-Hernandez et al., 2020; Lea et al., 2022). This fatigue can increase perceived exertion, impair movement quality, and diminish the overall training experience, potentially hindering long-term adherence in sedentary individuals who have lower tolerance for fatiguing exercise (Gonzalez-Hernandez et al., 2020; Lea et al., 2022; Jukic et al., 2020; Yang et al., 2017; Teychenne et al., 2010; Zhai et al., 2015; Allen et al., 2019; Gao et al., 2023). Existing evidence further suggests that cluster-style methods may improve tolerability by attenuating acute fatigue, particularly in less-trained populations (Oliver et al., 2015; Dello Iacono et al., 2020; Boffey et al., 2021). We hypothesize that more frequent intra-set rest may reduce fatigue accumulation and sessional stress, reflected by lower perceived exertion and smaller cortisol perturbation, thereby improving recovery and subsequent sleep. Accordingly, any psychological benefit is expected to arise indirectly through altered internal load, recovery, and sleep-related stress regulation rather than from a unique psychiatric mechanism of cluster sets.

Current evidence suggests that resistance training can improve sleep and mental-health outcomes, and that cluster-set strategies may enhance exercise tolerability (Gordon et al., 2020; O'Sullivan et al., 2023; Cunha et al., 2024; Herring and Meyer, 2024; Reid et al., 2010; Jukic et al., 2020; Kredlow et al., 2015). Nevertheless, it is still unclear whether rest-redistribution cluster sets provide additional benefits for mental health and sleep outcomes beyond traditional sets when overall training content, load progression, and total rest time are matched. This question is especially evident in sedentary young women. Although recent evidence indicates that structured exercise interventions can improve sleep parameters in sedentary young women, direct comparisons of resistance training set structures are scarce (Zhang et al., 2025).

Therefore, the present randomized controlled trial has three aims. First, it will compare traditional resistance training and cluster-set resistance training with a non-exercising control condition to determine their effects on mental health and sleep outcomes in sedentary young women. Second, it will examine whether rest-redistribution cluster sets confer additional benefits over traditional sets when training content, load progression, and total rest time are matched. Third, it will explore changes following the 8-week intervention in maximal strength, body composition, and blood biomarkers to provide exploratory information on physiological responses.

## 2 Methods

### 2.1 Design

**Figure 1:**
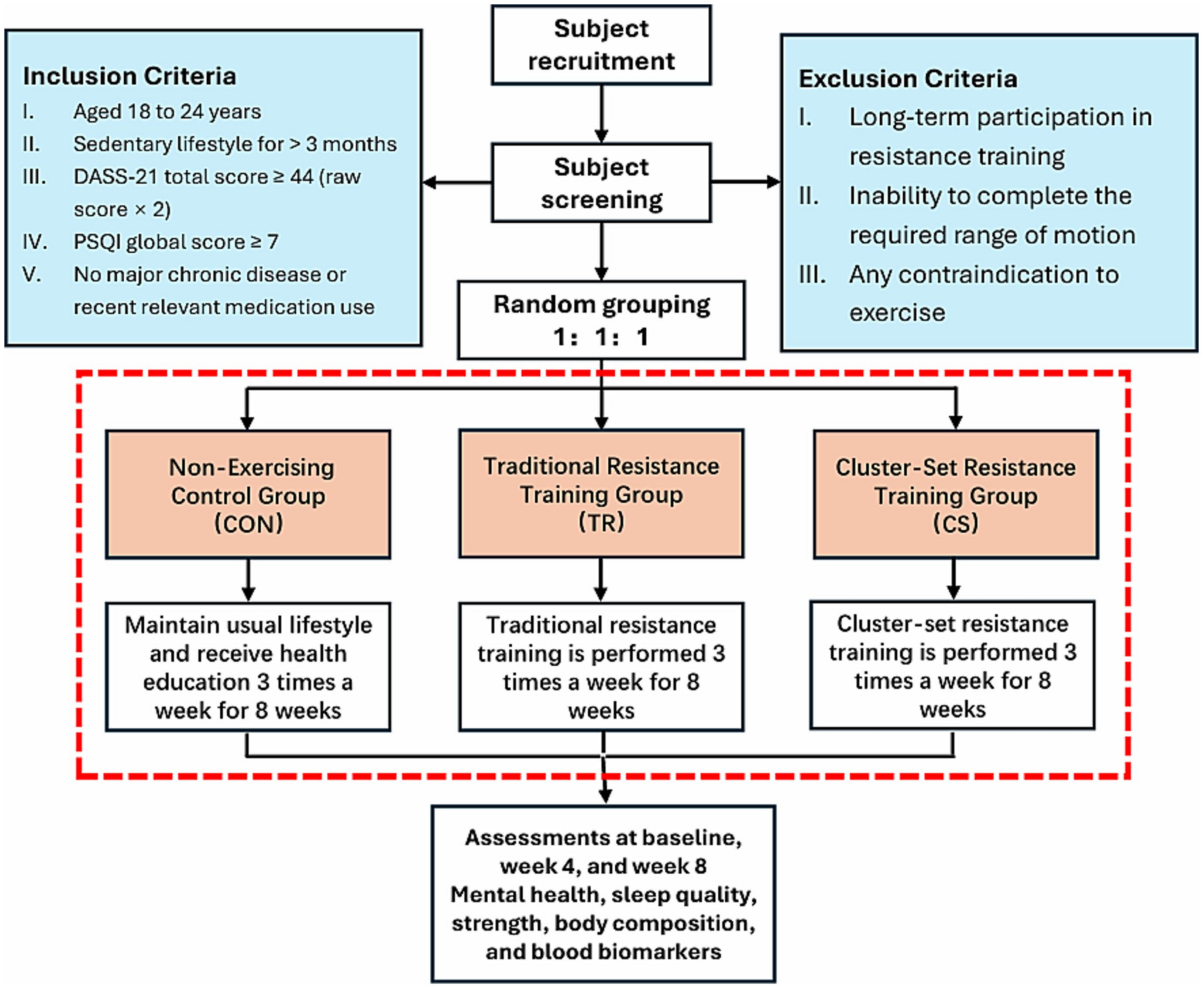
Flowchart of subject recruitment and study implementation.

This single-blind, three-arm, parallel-group randomized controlled trial will be conducted at Beijing Sport University. The study aims to compare the effects of a non-exercising control condition (CON), traditional resistance training (TR), and rest-redistribution cluster-set resistance training (CS) on sleep quality and mental health in sedentary female college students. Outcome assessors will be blinded to group allocation. The study flowchart is shown in Figure 1.

### 2.2 Participants

Participants will be sedentary female college students from universities in Beijing. They will be randomly assigned in a 1:1:1 ratio to CON, TR, or CS. We plan to enroll 48 participants (16 per group). The inclusion and exclusion criteria are as follows.

#### 2.2.1 Inclusion criteria

- Aged 18 to 24 years.
- No history of cardiovascular, hepatic, renal, or other major chronic diseases, and no current use within the past 3 months of medications that would contraindicate safe participation in resistance training.
- A sedentary lifestyle, defined as a daily sedentary time of >6 h and participation in moderate-intensity physical activity on fewer than 3 days per week (for at least 30 min per session) for over 3 months.
- Scores on the Depression, Anxiety and Stress Scales-21 Items (DASS-21) ≥ 44 (calculated by doubling the raw scores) (Lovibond and Lovibond, 1995).
- A Pittsburgh Sleep Quality Index (PSQI) global score of ≥ 7.0.

#### 2.2.2 Exclusion criteria

- An athlete or regular fitness enthusiast engaged in long-term resistance training.
- Inability to complete the full range of motion required by the intervention.
- Presence of any contraindication to exercise.

### 2.3 Sample size estimation

Although the final efficacy analyses will use linear mixed-effects models, sample size was estimated at the design stage using the repeated-measures ANOVA interaction framework in G*Power as a pragmatic approximation for a three-group repeated-assessment trial. The primary outcomes for sample size calculations are DASS-21 scores and PSQI scores. Two-sided tests with effect size *f* = 0.25, *α* = 0.05, and power (1-*β*) = 80% were used with three groups and three measurements. The minimum total sample size required for the study was determined to be 36 participants after sample size calculation with repeated measures analysis of variance (between-group-within-group interaction) using G * Power Software (version 3.1.9.7) (Kang, 2021). Considering a potential dropout rate of 20–25% based on our previous similar studies, we decided to recruit 48 eligible young women.

### 2.4 Recruitment

Sedentary female college students will be recruited from universities in Beijing through campus-wide advertisements and online announcements. Individuals expressing interest will first undergo a preliminary eligibility screening conducted by the research team. Those who meet the initial criteria will be invited to an on-site screening visit to further verify eligibility against the predefined inclusion and exclusion criteria.

Eligibility will be confirmed based on age (18–24 years), sedentary lifestyle characteristics, relevant medical history, and questionnaire-based thresholds, including a DASS-21 score ≥ 44 and a PSQI global score of ≥ 7.0. Exclusion criteria will be assessed concurrently, including long-term habitual resistance training (e.g., athletes or regular fitness enthusiasts), inability to complete the required range of motion, and any medical contraindication to exercise participation.

Prior to enrolment, eligible participants will receive a detailed explanation of the study procedures and will provide written informed consent. After completion of baseline assessments, participants will be randomly assigned in a 1:1:1 ratio to the non-exercising control group (CON), traditional resistance training (TR), or rest-redistribution cluster-set resistance training (CS). Recruitment will continue until the target sample size is achieved.

### 2.5 Randomization and blinding

After completion of baseline assessments and confirmation of eligibility, participants will be randomly allocated in a 1:1:1 ratio to the non-exercising control group (CON), traditional resistance training group (TR), or rest-redistribution cluster-set resistance training group (CS). The allocation sequence will be generated by an independent researcher who is not involved in participant recruitment, intervention delivery, outcome assessment, or statistical analysis, using a computer-generated permuted-block randomization scheme with variable block sizes. Variable block randomization was selected because the planned sample size is modest and approximate balance across the three groups should be maintained throughout enrolment, while undisclosed variable block sizes reduce the predictability of allocation. Stratification by baseline DASS-21 or PSQI score will not be used, because simultaneous stratification by multiple symptom measures in a small three-arm trial may create sparse strata and increase operational complexity without a clear gain in allocation efficiency. To ensure allocation concealment, group assignments will be placed in sequentially numbered, opaque, sealed envelopes and stored securely by the independent researcher. Study staff responsible for enrolment will confirm eligibility and complete baseline assessments before opening the next envelope in sequence, and these staff will have no prior access to the allocation sequence.

Given the nature of the exercise intervention, participants and exercise supervisors cannot be blinded to group allocation. However, this trial will adopt a single-blind design in which outcome assessors will remain blinded to group assignment throughout the study. Participants will be instructed not to disclose their allocation during outcome assessments, and assessment visits will be scheduled and conducted by staff members who are not involved in intervention supervision. Data will be recorded using coded participant identifiers, and the statistician will perform analyses using masked group labels until completion of the primary analyses. Unblinding will be permissible only when knowledge of group allocation is essential for participant safety or the management of a serious adverse event. Any request for unblinding will be reviewed by the principal investigator, who, if necessary, will obtain the allocation information from the independent researcher responsible for the randomization sequence. If unblinding occurs, only the minimum necessary personnel will be informed, and the reason and timing will be documented in the trial records.

### 2.6 Informed consent

The trial will be conducted in accordance with the Declaration of Helsinki and applicable institutional ethics requirements (World Medical A, 2013). The study protocol was approved by the Ethics Committee of the Exercise Science Laboratory at Beijing Sport University (no. 2025422H) and prospectively registered with the Chinese Clinical Trial Registry (no. ChiCTR2500110077; registered on September 29, 2025). Eligible participants will receive written and verbal information describing study aims, procedures, potential risks and benefits, confidentiality protections, and their rights. Written informed consent will be obtained before any study-related assessments. Data will be coded and stored on password-protected, encrypted systems accessible only to authorized study personnel. Participants may withdraw at any time without penalty, and any new information that could influence willingness to continue will be communicated promptly to support ongoing informed consent.

If a participant withdraws after randomization, she will not be reassigned to another study arm; her status will be recorded as withdrawn. With permission, we will invite (but not require) a brief exit interview to document reasons for withdrawal and to inform future protocol refinement. Participants who cannot be contacted for the final assessment, regardless of their intervention status, will be recorded as lost to follow-up.

### 2.7 Intervention

This is a three-arm, parallel-group trial comparing a non-exercising control group (CON), traditional resistance training (TR), and rest-redistribution cluster-set resistance training (CS). The intervention period will last 8 weeks. TR and CS will complete three supervised training sessions per week (approximately 48–72 h apart). Training exercises will be identical between TR and CS; only set structure and rest allocation will differ. Participants in CON will not participate in any structured resistance-training sessions and will be instructed to maintain their usual lifestyle and physical-activity habits during the study period.

#### 2.7.1 Session structure and general procedures

##### 2.7.1.1 Step 1: warm-up exercises

Each supervised training session for TR and CS will include: (1) standardized warm-up, (2) resistance training, and (3) standardized cool-down. Coaches will provide standardized technique cues and ensure correct movement execution. Rest intervals will be timed using a stopwatch.

##### 2.7.1.2 Step 2: resistance training

Participants in TR and CS will perform the same five exercises in the same order: supine abdominal curl, plank, wall sit, squat, and deadlift. The only planned difference between TR and CS is the set configuration and distribution of rest intervals.

##### 2.7.1.3 Step 3: cool-down

After training, participants will complete a standardized 10-min cool-down. A 3–5-min brisk walk will be used to reduce heart rate, followed by static stretching of major muscle groups (hamstrings, quadriceps, gluteals, and calves). Participants will then perform cat–cow and child’s pose stretches to relax the core and lower back. Finally, foam rolling will be applied to commonly tight areas (e.g., quadriceps, iliotibial band, gluteals, hamstrings, and calves) to reduce perceived stiffness and support recovery.

##### 2.7.1.4 Procedures for CON

Participants in CON will not attend training sessions. They will be asked to maintain their usual lifestyle, avoid initiating a new structured exercise program, and complete the same outcome assessments as the training groups at baseline, week 4, and week 8. To minimize differential attention and support retention, CON participants will receive the same brief sleep-health education provided at baseline to all participants and will be contacted weekly to record major changes in physical activity, sleep medication use, or health status. After completion of the study, CON participants will be offered access to the supervised resistance-training program if desired.

#### 2.7.2 Intervention arms and set-structure definitions

For each exercise, total rest time will be matched at 240 s across groups to improve comparability. The two set structures are defined as follows:

- TR (traditional sets): 3 sets per exercise with between-set rest of 120 s (two rest periods per exercise; total rest = 240 s).
- CS (rest-redistribution cluster sets): each traditional set is partitioned into two clusters. A brief intra-set rest (20 s) is inserted after cluster 1. Between-set rest is reduced to 90 s (two rest periods per exercise; total between-set rest = 180 s). The remaining 60 s rest is reallocated as intra-set rest (3 sets × 20 s intra-set rest per set = 60 s), such that total rest per exercise remains 240 s. The number of micro-sets differs by exercise (Table 1).

**Table 1:** Resistance-training prescription and set structure by study arm.

Exercise	TR	CS	Total rest
Wall sit	3 × 60 s holds; 120 s rest between sets.	3 sets; each set: 2 × 30 s holds with 20 s intra-set rests; 90 s between sets.	240 s
Plank	3 × 60 s holds; 120 s rest between sets.	3 sets; each set: 2 × 30 s holds with 20 s intra-set rests; 90 s between sets.	
Supine abdominal curl	3 × 12 reps; 120 s rest between sets.	3 sets; each set: clusters 6–6 reps with 20 s intra-set rests; 90 s between sets.	
Squat	3 × 8 reps; 120 s rest between sets.	3 sets; each set: clusters 4–4 reps with 20 s intra-set rests; 90 s between sets.	
Deadlift	3 × 8 reps; 120 s rest between sets.	3 sets; each set: clusters 4–4 reps with 20 s intra-set rests; 90 s between sets.	

#### 2.7.3 Load progression and monitoring

Squat and deadlift loads will be prescribed relative to each participant’s baseline one-repetition maximum (1RM) and progressed linearly: 60% 1RM in weeks 1–2, 65% 1RM in weeks 3–4, 67.5% 1RM in weeks 5–6, and 70% 1RM in weeks 7–8. Coaches will record attendance, completion of prescribed sets and rests, and any adverse events at each session. For CON, weekly contacts will record major changes in health status and physical activity. In addition to the standardized external-load prescription, internal training load will be monitored using the session rating of perceived exertion (sRPE) method. Following the standardized cool-down, participants will report a global sRPE using the modified Borg CR-10 scale, and session training load will be calculated as sRPE × session duration (min) and expressed in arbitrary units (Singh et al., 2007).

### 2.8 Strategies to improve adherence to interventions

Adherence-support procedures were informed by the COM-B framework, which conceptualizes sustained exercise behavior as dependent on capability, opportunity, and motivation. Within this framework, standardized instruction and technique guidance were intended to support capability, supervised scheduling and follow-up after missed sessions were intended to support opportunity, and sleep-health education, progress feedback, and supportive communication were intended to support motivation. To encourage adherence and minimize attrition, we will: (1) explain participant responsibilities during recruitment and consent and establish open communication channels; (2) provide all participants (including CON) with a brief standardized education session on sleep health and the potential benefits of evidence-based resistance training; (3) monitor training attendance and protocol fidelity weekly in TR and CS, with follow-up contact within 48 h after any unexcused absence; and (4) provide periodic feedback on participation progress and reinforce engagement through supportive communication. For CON, weekly check-ins will focus on retention and documenting potential contamination (e.g., initiation of a new exercise program).

### 2.9 Standardization procedures before repeated assessments

To reduce measurement variability across baseline, week 4, and week 8 assessments, repeated testing will be conducted under standardized conditions whenever feasible (Ward, 2019; Tinsley et al., 2022). Participants will be assessed at approximately the same time of day across visits, by the same assessors, using the same equipment, device settings, and testing order. They will be instructed to avoid vigorous physical activity, alcohol, and non-essential caffeine for 24 h before each assessment visit and to maintain their usual sleep schedule on the preceding night. Menstrual-cycle phase and major academic stressors (eg, examinations) will be recorded at each assessment.

For body composition and blood biomarker assessments, participants will attend the laboratory in the morning after an overnight fast, after bladder voiding, and before any exercise on that day. For strength testing, participants will be asked to refrain from strenuous exercise for at least 48 h before testing and will complete the same standardized warm-up and test procedures at each time point. For actigraphy-based sleep assessment, the same wear instructions, monitoring duration, and scoring procedures will be applied at each assessment point (Ancoli-Israel et al., 2015).

### 2.10 Safety monitoring and adverse event reporting

This study will employ a prespecified adverse event monitoring framework. Any unfavorable medical event occurring during the study period that is related to study participation or temporally associated with the study intervention will be defined as an adverse event (AE). Events resulting in hospitalization, serious injury, persistent functional impairment, or any other important medical risk will be classified as serious adverse events (SAEs).

In the TR and CS groups, coaches or research staff will proactively inquire about symptoms or discomfort before and after each training session and document any training-related events. In the CON group, changes in health status will be assessed during weekly follow-up contacts. For all AEs, the nature of the event, onset time, duration, severity, management, outcome, and relatedness to the intervention will be recorded. Participants requiring temporary cessation of training or further medical evaluation will suspend participation until reviewed by the principal investigator. Any SAE will be reported promptly in accordance with ethics committee and institutional requirements.

### 2.11 Outcome

#### 2.11.1 Primary outcomes

The primary outcomes are DASS-21 total score and PSQI global score. DASS-21 was selected as the primary mental-health outcome, and PSQI was selected as the primary sleep outcome. For both co-primary outcomes, the confirmatory time point of interest will be week 8, with week 4 serving as an intermediate follow-up assessment.

##### 2.11.1.1 Depression, anxiety, and stress scales-21 items (DASS-21)

DASS-21 is a 21-item self-report questionnaire that quantifies symptoms of depression, anxiety, and stress. Items are organized into three 7-item subscales and rated from 0 to 3 to reflect symptom severity/frequency. Subscale scores (and an overall total score) are calculated by summing item responses; higher scores indicate greater symptom burden. The instrument has been widely applied in research and community samples and shows acceptable reliability and construct validity (Zhang et al., 2024).

##### 2.11.1.2 Pittsburgh sleep quality index (PSQI)

Subjective sleep quality will be assessed with the PSQI, which evaluates overall sleep quality and common sleep disturbances over the previous month. The PSQI contains 19 self-rated items that generate seven component scores (subjective sleep quality, sleep latency, sleep duration, habitual sleep efficiency, sleep disturbances, use of sleep medication, and daytime dysfunction). Each component is scored 0–3 and summed to yield a global score from 0 to 21, with higher scores indicating poorer sleep. A global score >5 is often used to indicate clinically relevant sleep disturbance (Buysse et al., 1989). For this study, the PSQI validated Chinese version will be used.

#### 2.11.2 Secondary outcomes

The secondary outcomes provide complementary characterization of POMS total mood disturbance, actigraphy-derived sleep parameters, participants’ strength, body composition, and biological pathways potentially linking resistance training to sleep and mental health. The schedule of enrolment, interventions, and assessments are shown in Table 2.

**Table 2:** The schedule of enrolment, interventions, and assessments.

Type of research	Data collection component	Content	Baseline (week 0)	Week 4	Week 8
Quantitative research	Eligibility screening and informed consent	Assess criteria; obtain written informed consent; randomize participants (1:1:1)	✓		
	Mental health	DASS-21; POMS	✓	✓	✓
	Sleep outcomes	Wrist-worn accelerometer; PSQI	✓	✓	✓
	Strength	1RM back squat; 1RM conventional deadlift	✓	✓	✓
	Biomarkers	High-sensitivity C-reactive protein; cortisol; serotonin	✓	✓	✓
	Anthropometrics	Weight/height/ body mass index; waist/hip/waist-to-hip ratio; Bioelectrical impedance analysis	✓	✓	✓
	Adherence and safety monitoring	TR/CS: attendance, completion, Adverse events each session; CON: weekly contacts record changes	Continuous/weekly monitoring		

##### 2.11.2.1 Profile of mood states (POMS)

The POMS is a self-report inventory designed to capture recent mood states. The 65 adjective items form six domains (tension, depression, anger, vitality, fatigue, and confusion). Participants indicate how strongly each adjective described their feelings over the past week (including the day of assessment) on a 5-point scale from 1 (none) to 5 (extremely). Total mood disturbance is computed by summing the negative domains, subtracting the vitality score, and adding the standard constant; higher values reflect greater overall emotional distress (Schmalbach et al., 2025).

##### 2.11.2.2 Actigraphy-derived sleep parameters

Objective sleep will be assessed using wrist-worn actigraphy (e.g., ActiGraph). Sleep parameters will be derived using validated actigraphy algorithms and will include indicators such as total sleep time, sleep efficiency, sleep onset latency, wake after sleep onset, and the number/duration of awakenings (Ancoli-Israel et al., 2015).

##### 2.11.2.3 Maximal dynamic strength

Maximal lower-body dynamic strength will be quantified via 1RM testing for the back squat and conventional deadlift, defined as the highest load that can be lifted once through the full range of motion with proper technique. Testing will follow standardized warm-up and incremental loading procedures with adequate rest between attempts; 1RM testing is considered a gold-standard field measure of maximal dynamic strength and demonstrates good-to-excellent test–retest reliability across a wide range of populations and exercises (Grgic et al., 2020). To enhance comparability across repeated measurements, 1RM testing will be performed under the same standardized warm-up, assessor supervision, and pre-test restrictions at each time point (Grgic et al., 2020).

##### 2.11.2.4 Anthropometric measurements and body composition

The participants will complete anthropometric assessments barefoot and wearing light clothing. Body mass and height will be recorded using calibrated equipment, and BMI will be calculated as weight (kg) divided by height squared (m^2^). Waist circumference will be measured at the level of the umbilicus and hip circumference at the widest point; each will be taken twice and averaged, and the waist-to-hip ratio will be computed as waist/hip. Body composition will be assessed via bioelectrical impedance analysis using a Tanita TBF-418B device (Tanita Corporation, Tokyo, Japan). Participants will stand on the analyzer with appropriate foot contact on the electrodes; the device applies a low-amplitude alternating current (0.8 mA, 50 kHz) and estimates whole-body and trunk fat mass and percentage fat based on impedance. Reported in-vivo reproducibility for this system is 1.54% (BF), 1.85% (%BF), 2.50% (TF), and 2.79% (%TF) (Zhang et al., 2014). To improve repeatability, body composition assessments will be standardized with respect to time of day, fasting status, bladder voiding, and pre-assessment physical activity.

##### 2.11.2.5 Blood biomarkers

To provide an exploratory biological framework for the secondary outcomes, hs-CRP, cortisol, and serotonin were selected *a priori* as peripheral markers representing three biologically plausible pathways through which resistance training may influence mental health and sleep, namely low-grade systemic inflammation, neuroendocrine stress regulation, and serotonergic signaling. Specifically, hs-CRP was included as an index of low-grade systemic inflammation, which may be elevated in sedentary individuals and has been associated with depressive symptoms and sleep disturbance (Pearson et al., 2003; Howren et al., 2009). Cortisol was selected to reflect hypothalamic–pituitary–adrenal axis activity and circadian stress regulation, given its pronounced diurnal rhythm and sensitivity to chronic stress, both of which are relevant to sleep and psychological well-being (Liu, 2024). Serotonin was included as an exploratory peripheral marker related to mood regulation and sleep–wake control, and as a biochemical precursor of melatonin; altered serotonergic activity has been implicated in depression, anxiety, and sleep dysregulation (Monti, 2011; Yohn et al., 2017). Collectively, these biomarkers were intended to provide exploratory insight into inflammatory, neuroendocrine, and serotonergic pathways potentially relevant to intervention-related changes in mental health and sleep, rather than to serve as definitive mechanistic mediators.

Venous blood samples will be collected at baseline, week 4, and week 8 between 07:00 and 09:00 after an overnight fast of at least 8 h. To minimize acute exercise-related perturbations, blood sampling in the TR and CS groups will be scheduled 48–72 h after the most recent training session. Participants will be asked to avoid vigorous physical activity outside the intervention, alcohol, and caffeine for 24 h before sampling. Samples will be collected after a standardized seated rest period and processed according to a pre-specified analyte-specific protocol. After centrifugation, aliquots will be stored at −80 °C until batch analysis. All assays will be performed in the same laboratory by personnel blinded to group allocation, and samples from the same participant will be analyzed within the same assay batch whenever feasible. Intra- and inter-assay coefficients of variation will be recorded and reported.

### 2.12 Statistical analysis

All statistical analyses will be performed using SPSS version 26.0 and R software. Except for multiplicity-adjusted analyses of the co-primary outcomes, a two-sided *p*-value of <0.05 will be considered statistically significant. Continuous variables will be summarized as mean ± standard deviation (SD) if approximately normally distributed, or as median (interquartile range) otherwise. Categorical variables will be summarized as counts and percentages. Baseline characteristics will be described by group. The co-primary outcomes will be the DASS-21 total score and the PSQI total score.

The primary analysis will follow the intention-to-treat principle and will use linear mixed-effects models for repeated measures, given the longitudinal design and the possibility of incomplete follow-up (Krueger and Tian, 2004). For each continuous outcome, fixed effects will include group (CON, TR, and CS), time (Baseline, Week 4, and Week 8; treated as a categorical variable), and the group × time interaction, with participant included as a random effect to account for within-subject correlation across repeated assessments. For the co-primary outcomes, the primary inferential tests will focus on the group × time effects, with Holm-Bonferroni correction applied to control multiplicity across the two co-primary outcomes. For each co-primary outcome, the primary contrast will be the between-group difference in change from baseline to Week 8 estimated from the mixed-effects model. Prespecified pairwise comparisons among CON, TR, and CS, including CON versus TR, CON versus CS, and TR versus CS, will be conducted using model-estimated marginal means with adjustment for multiple comparisons. Analyses of Week 4 outcomes and secondary outcomes will be considered exploratory.

Missing outcome data will not be routinely handled by multiple imputation in the primary analysis. Instead, the mixed-model framework will use maximum likelihood estimation based on all available repeated measurements, which provides valid inference under a missing-at-random assumption. To improve transparency, the amount, patterns, and reasons for missing data will be summarized by group and time point (Pugh et al., 2022). If missing data for the primary outcomes are non-negligible, sensitivity analyses using multiple imputation and, where appropriate, an alternative missing-data framework (eg, pattern-mixture analysis) will be performed to assess the robustness of the findings to departures from the primary assumption.

Model assumptions will be examined using residual diagnostics. Variables with marked skewness, particularly biomarker measures, may be transformed as appropriate before modelling. Results will be reported as model-based estimated mean differences or regression coefficients with 95% confidence intervals. Standardized effect sizes will also be presented for key between-group contrasts where appropriate.

### 2.13 Study management

#### 2.13.1 Data collection

All participants will complete the relevant questionnaires at baseline and at each follow-up visit. The online questionnaires will be distributed in the form of QR codes via the Questionnaire Star platform, which participants will scan to complete and submit electronically. Baseline physical activity will be characterized by self-report, and major changes in non-intervention physical activity during follow-up will be recorded.

#### 2.13.2 Storage and archiving of data

The research team will store all study-related data (e.g., baseline and follow-up questionnaires, randomization records, and follow-up information logs) and documents (e.g., participants’ informed consent forms and accelerometer data files) in a dedicated study database in a standardized format. After the trial is completed, all source data and files will be organized and archived in accordance with applicable laws and regulations. Access to the database will be permission-based and requires authorization from the investigators; visitor information and the purpose of access will be mandatorily recorded to prevent unauthorized access and misuse of data. Any planned or unplanned unblinding events will be recorded in a dedicated study log and archived with the trial documentation.

Study data will be stored in de-identified form using coded participant identifiers, with the participant-identification linkage file stored separately in a restricted-access folder. Access rights will be role-based and limited to authorized study personnel. Data entry, verification, and any subsequent corrections will be documented to maintain an audit trail.

Given the single-centre, investigator-initiated, minimal-risk design of this trial, no formal independent external audit is planned. Instead, the principal investigator will conduct periodic internal monitoring of protocol adherence, data completeness, adverse-event documentation, and essential trial records throughout the study.

## 3 Discussion

The central scientific question of this trial is whether resistance training improves sleep quality and mental health in sedentary young women compared with a non-exercising control condition, and whether rest-redistribution cluster sets provide additional benefits over traditional set structures when training volume, load progression, and total rest time are matched. Sedentary behavior is associated with poorer sleep and increased risk of stress, anxiety, and depressive symptoms. Although traditional resistance training can improve physical and mental health outcomes, protocols that induce high levels of metabolic stress and perceived exertion may reduce exercise enjoyment and long-term adherence in sedentary populations.

Cluster-set training with rest-redistribution offers a promising approach to balance training stimulus with reduced intra-set fatigue. By inserting brief intra-set rests and modestly reducing between-set rest, the structure is designed to preserve movement quality and lower perceptual strain compared with traditional sets, while maintaining equivalent overall training load. This trial will examine whether such modifications are associated with differences in sleep and mental-health outcomes, potentially in relation to training experience, adherence, and selected physiological responses.

By prioritizing DASS-21 and PSQI as co-primary outcomes, and treating POMS and actigraphy-derived sleep parameters as supportive secondary outcomes, this study aims to provide a focused assessment of psychological distress and subjective sleep quality while retaining broader characterization of mood, objective sleep, strength, body composition, and biomarkers. The inclusion of a non-exercising control group strengthens causal inference by accounting for natural temporal variation, such as academic stress, and measurement familiarity effects common among college students.

Several limitations should be acknowledged. First, although the planned sample size was estimated for the co-primary outcomes, it remains limited. This trial is expected to detect moderate group-by-time interaction effects for the two co-primary outcomes, namely the DASS-21 total score and the PSQI global score, but may be underpowered to detect small effects in secondary outcomes, such as actigraphy-derived sleep parameters and blood biomarkers. Therefore, findings for secondary outcomes and biomarkers will be interpreted as exploratory, with emphasis placed on the direction and magnitude of estimated effects and their 95% confidence intervals rather than solely on statistical significance. Second, the 8-week intervention duration is sufficient to detect short-term changes but may not capture the long-term maintenance of benefits; future studies with extended follow-up are warranted. In addition, the actigraphy measures in this protocol are intended to characterize sleep continuity rather than polysomnographic sleep architecture, and the biomarker panel should be interpreted as exploratory because cortisol is strongly time-dependent and peripheral serotonin is sensitive to pre-analytical handling. Third, because this is a single-centre trial recruiting sedentary female college students from universities in Beijing, the findings may not generalize directly to men, non-student populations, other geographic or socioeconomic contexts, or women with different hormonal characteristics. Finally, although additional standardization and monitoring procedures have been introduced for factors such as caffeine exposure, menstrual-cycle phase, academic stress, and non-intervention physical activity, residual confounding cannot be fully excluded in this pragmatic field-based trial.

## Dissemination

Research findings will be disseminated through academic conferences and scientific journals. Data will be collected electronically and stored in secure databases accessible only to the research team. With participant consent, anonymized research data may be used as comparative material for secondary analysis in future studies.
