# Supplementary material for: The effects of cluster-set resistance training on mental health and sleep quality in sedentary young women: protocol for a randomized controlled trial
Source: Front Psychol. 2026 Jun 5;17:1846281. doi: 10.3389/fpsyg.2026.1846281 (PMC13278862; doi:10.3389/fpsyg.2026.1846281)
Supplement: Supplementary file 1 [file Supplementary_file_1.DOCX]

**Supplementary Appendix S1
SPIRIT 2013 checklist**

**Journal-facing note.** This appendix maps the revised manuscript to the SPIRIT 2013 checklist using manuscript section headings rather than page numbers, because pagination may change during final editing. Items that are not applicable are marked N/A.

| **Item** | **SPIRIT 2013 checklist item** | **Manuscript location in revised draft** | **Notes / N/A** |
| --- | --- | --- | --- |
| 1 | Descriptive title identifying the study design, population, interventions | Title page |  |
| 2a | Trial identifier and registry name | Abstract; Informed consent; Ethics statement |  |
| 2b | WHO Trial Registration Data Set items | Abstract; Design; Participants; Recruitment; Outcomes | Core items distributed across protocol |
| 3 | Protocol version and date | Not specified in the current protocol | Protocol version identifier is not specified in the current protocol. |
| 4 | Sources and types of support | Funding |  |
| 5a | Names, affiliations, and roles of protocol contributors | Title page; Author Contributions |  |
| 5b | Name and contact information for trial sponsor | Corresponding author details / investigator-initiated academic study | Investigator-initiated academic study; no external funding is reported. |
| 5c | Role of study sponsor and funders | Funding | No external funding is reported |
| 5d | Coordinating centre / oversight roles | Not specified in the current protocol | No steering committee or endpoint adjudication committee is described for this single-centre investigator-initiated trial. |
| 6a | Background and rationale, including relevant studies | Introduction |  |
| 6b | Explanation for choice of comparators | Introduction; Procedures for CON; 2.7.2 Intervention arms |  |
| 7 | Specific objectives or hypotheses | Introduction |  |
| 8 | Trial design, allocation ratio, and framework | Design |  |
| 9 | Study setting and sites/countries | Design; Participants; Recruitment |  |
| 10 | Eligibility criteria | Participants |  |
| 11a | Interventions with sufficient detail for replication | 2.7.1–2.7.3; Table 1 |  |
| 11b | Criteria for discontinuing or modifying interventions | Informed consent | Temporary cessation of training and withdrawal procedures are described. |
| 11c | Strategies to improve adherence and procedures for monitoring adherence | Strategies to improve adherence; 2.7.3 Load progression and monitoring |  |
| 11d | Relevant concomitant care and permitted/prohibited interventions | Intervention; Procedures for CON; eligibility criteria | Participants in CON are asked to avoid initiating a new structured exercise program; changes in physical activity, sleep medication use, and health status are monitored. |
| 12 | Primary, secondary, and other outcomes, with metrics and time points | 2.11.1–2.11.2 Outcome; Table 2 |  |
| 13 | Participant timeline (diagram recommended) | Figure 1; Table 2 |  |
| 14 | Sample size and assumptions | Sample size estimation |  |
| 15 | Recruitment strategies | Recruitment |  |
| 16a | Method of generating allocation sequence | Randomization and blinding |  |
| 16b | Allocation concealment mechanism | Randomization and blinding | Allocation concealment is implemented using sequentially numbered, opaque, sealed envelopes. |
| 16c | Who generates sequence, enrols, and assigns participants | Recruitment; Randomization and blinding | Allocation sequence generation and enrolment procedures are described. |
| 17a | Who will be blinded and how | Randomization and blinding |  |
| 17b | Permissible unblinding and procedure | Randomization and blinding; 2.13.2 Storage and archiving of data | Permissible unblinding conditions and documentation procedures are described. |
| 18a | Plans for assessment and data collection / data quality | Outcome sections; 2.13.1 Data collection; Standardization procedures |  |
| 18b | Plans to promote retention and complete follow-up | Informed consent; Strategies to improve adherence; Procedures for CON |  |
| 19 | Data management (entry, coding, security, storage, data-quality processes) | Informed consent; 2.13.1; 2.13.2 | De-identification, role-based access, data verification, and audit-trail procedures are described. |
| 20a | Statistical methods for primary and secondary outcomes | Statistical analysis | Linear mixed-effects models are specified as the primary analytic framework. |
| 20b | Methods for additional analyses | Statistical analysis | Sensitivity analyses for non-negligible missing primary outcome data are described. |
| 20c | Analysis population and methods for missing data | Statistical analysis | Intention-to-treat analysis and maximum likelihood estimation using all available repeated measurements are described. |
| 21a | Data monitoring committee or explanation if none | Not specified in the current protocol | Not specified in the current protocol. |
| 21b | Interim analyses / stopping guidelines | Not specified in the current protocol | Not specified in the current protocol. |
| 22 | Plans for collecting, assessing, reporting, and managing harms | 2.7.3 Load progression and monitoring; Safety monitoring and adverse event reporting; Table 2 | AE/SAE definitions, documentation, management, and reporting procedures are described. |
| 23 | Frequency and procedures for auditing trial conduct | 2.13.2 Storage and archiving of data | No formal independent external audit is planned; periodic internal monitoring by the principal investigator is described. |
| 24 | Plans for REC/IRB approval | Informed consent; Ethics statement |  |
| 25 | Plans for communicating protocol modifications | Not specified | Not specified in the current protocol. |
| 26a | Who will obtain informed consent and how | Recruitment; Informed consent |  |
| 26b | Additional consent for use of data/specimens in ancillary studies | Dissemination; biomarker section | Use of anonymized research data for future secondary analysis is described as conditional on participant consent. |
| 27 | Confidentiality protections | Informed consent; 2.13.2 |  |
| 28 | Financial and other competing interests | Conflict of Interest |  |
| 29 | Who will have access to final trial dataset | 2.13.2 Storage and archiving of data | Access is restricted to authorized study personnel/the research team. |
| 30 | Ancillary and post-trial care / compensation for harm | Procedures for CON; Safety monitoring and adverse event reporting | Post-trial access to the supervised resistance-training program for CON participants is described; compensation for harm is not specified. |
| 31a | Plans to communicate trial results | Dissemination |  |
| 31b | Authorship eligibility and professional writers | Author Contributions; Generative AI statement | Author contributions are described; no generative AI use is declared. |
| 31c | Plans for public access to protocol, dataset, and statistical code | Dissemination | Dissemination through conferences and journals is described; public access to the full dataset and statistical code is not specified. |
| 32 | Model consent form and related documentation | Informed consent | Model consent form is not included as supplementary material. |
| 33 | Plans for collection, lab evaluation, storage, and future use of biological specimens | Blood biomarkers section | Collection, processing, storage, and laboratory analysis of blood samples are described; long-term biobanking or future use beyond the specified biomarker analyses is not specified. |
